# Phylodynamics of deer tick virus in North America

**DOI:** 10.1093/ve/vead008

**Published:** 2023-01-27

**Authors:** Rebekah J McMinn, Rose M Langsjoen, Andrei Bombin, Rebecca M Robich, Erick Ojeda, Erica Normandin, Heidi K Goethert, Charles B Lubelczyk, Elizabeth Schneider, Danielle Cosenza, Molly Meagher, Melissa A Prusinski, Pardis C Sabeti, Robert P Smith, Sam R Telford, Anne Piantadosi, Gregory D Ebel

**Affiliations:** Department of Microbiology, Immunology, and Pathology, Colorado State University, Fort Collins, CO 80523, USA; Department of Pathology and Laboratory Medicine, Emory University School of Medicine, Atlanta, GA 30307, USA; Department of Medicine, Division of Infectious Diseases, Emory University School of Medicine, Atlanta, GA 30307, USA; Maine Health Institute for Research, Scarborough, ME 04074, USA; Department of Pathology and Laboratory Medicine, Emory University School of Medicine, Atlanta, GA 30307, USA; Broad Institute of MIT and Harvard, Cambridge, MA 02142, USA; Center for Systems Biology, Harvard Medical School, Boston, MA 02115, USA; Department of Infectious Disease and Global Health, Tufts University, North Grafton, MA 01536, USA; Maine Health Institute for Research, Scarborough, ME 04074, USA; Maine Health Institute for Research, Scarborough, ME 04074, USA; Maine Health Institute for Research, Scarborough, ME 04074, USA; Maine Health Institute for Research, Scarborough, ME 04074, USA; Bureau of Communicable Disease Control, New York State Department of Health, Albany, NY 12237, USA; Broad Institute of MIT and Harvard, Cambridge, MA 02142, USA; Department of Organismic and Evolutionary Biology, Harvard University, Cambridge, MA 02138, USA; Department of Immunology and Infectious Diseases, Harvard School of Public Health, Boston, MA 02115, USA; Howard Hughes Medical Institute, Chevy Chase, MD 20815, USA; Maine Health Institute for Research, Scarborough, ME 04074, USA; Department of Infectious Disease and Global Health, Tufts University, North Grafton, MA 01536, USA; Department of Pathology and Laboratory Medicine, Emory University School of Medicine, Atlanta, GA 30307, USA; Department of Medicine, Division of Infectious Diseases, Emory University School of Medicine, Atlanta, GA 30307, USA; Department of Microbiology, Immunology, and Pathology, Colorado State University, Fort Collins, CO 80523, USA

**Keywords:** tick-borne flavivirus, emerging virus, phylodynamics, Powassan virus, deer tick virus

## Abstract

The burden of ticks and the pathogens they carry is increasing worldwide. Powassan virus (POWV; Flaviviridae: *Flavivirus*), the only known North American tick-borne flavivirus, is of particular concern due to rising cases and the severe morbidity of POWV encephalitis. Here, we use a multifaceted approach to evaluate the emergence of the II POWV lineage, known as deer tick virus (DTV), in parts of North America where human cases occur. We detected DTV-positive ticks from eight of twenty locations in the Northeast USA with an average infection rate of 1.4 per cent. High-depth, whole-genome sequencing of eighty-four POWV and DTV samples allowed us to assess geographic and temporal phylodynamics. We observed both stable infection in the Northeast USA and patterns of geographic dispersal within and between regions. A Bayesian skyline analysis demonstrated DTV population expansion over the last 50 years. This is concordant with the documented expansion of *Ixodes scapularis* tick populations and suggests an increasing risk of human exposure as the vector spreads. Finally, we isolated sixteen novel viruses in cell culture and demonstrated limited genetic change after passage, a valuable resource for future studies investigating this emerging virus.

## 1. Introduction

Powassan virus (POWV) is an emerging tick-borne flavivirus endemic to North America and Far Eastern Russia. POWV can cause severe encephalitis leading to permanent neurological sequelae in 50 per cent of patients and death in 10 per cent (Ebel 2010; Krow-Lucal et al. 2018). Reported human cases in the USA have increased from 26 (1999 to 2009) to 186 (2010 to 2020) (Centers for Disease Control and Prevention 2023). Furthermore, the detection of POWV-neutralizing antibodies in Northeast hunter-killed deer increased from 4 per cent in 1979 to 91 per cent in 2010 (Nofchissey et al. 2013).

POWV is the only North American member of the Flavivirus tick-borne encephalitis serogroup, first identified in 1958 in a patient with encephalitis in Powassan, ON, Canada (McLean and Donohue 1959). The canonical I POWV lineage has been estimated to diverge from the European tick-borne encephalitis complex between 2,000 and 6,000 years ago, around the time the Bering Land Bridge between Alaska and Far Eastern Russia disappeared, effectively limiting migration between Russia and North America (Bondaryuk et al. 2021). In 1995, a second lineage of the virus, deer tick virus (DTV), was identified in ticks in the Northeast USA (Telford et al. 1997) and subsequently detected in Wisconsin 2 years later (Ebel et al. 1999). While lineage I POWV is typically found in nidicolous, host species-specific ticks such as *Ixodes cookei* and *I. marxi*, lineage II DTV is mainly found in human-biting *I. scapularis* ticks, thus posing a significant risk to humans (Ebel 2010). However, *I. cookei* and *I. marxi* have been documented to feed on humans, albeit infrequently (Rand et al. 2007).

Given the rise of human cases and expansion of *I. scapularis* ticks and their associated pathogens, we sought to gain a better understanding of DTV emergence in North America. Here, we report DTV prevalence in ticks from a multistate collection study in 2019 and isolation of diverse viruses in cell culture for in-depth studies. We present whole-genome, time-scaled phylogenic analyses of DTV in North America using 108 published and newly sequenced isolates that represent a broad geographic and temporal range.

## 2. Methods

### 2.1 Tick collection and screening

In the spring and fall of 2019, host-seeking *I. scapularis* ticks were collected during surveys of vegetation using square-meter white flannel drags (Salomon, Hamer, and Swei 2020). Survey sites were selected based on guidance from the New York State Department of Health, Maine Health Institute for Research, and Tufts University. Ticks were kept alive in vented 5-dram styrene vials at room temperature or 4°C with a paper towel moistened with water for up to 5 days before being moved to a 95 per cent relative humidity chamber at 9°C until processed. Ticks were surface sterilized in 3 per cent hydrogen peroxide and phosphate-buffered saline (PBS) for 20 sec each. Ticks were then bead homogenized for 2 min at 24 hertz in 200 μl PBS supplemented with 20 per cent fetal bovine serum (FBS), 1 per cent penicillin/streptomycin, 2.5 mg/ml amphotericin B, and 50 mg/ml gentamicin and finally centrifuged at 10,000 rcf for 5 min at 4°C. Homogenates were pooled in groups of five, and ribonucleic acid (RNA) was extracted using the Omega viral DNA/RNA kit on a KingFisher robotics platform. Quantitative reverse-transcriptase polymerase chain reaction (qRT-PCR) was performed on homogenate pools using the following primers directed to the *NS5* gene: GGCCATGACAGACACAACAGCGTTTG (forward) and GAGCGCTCTTCATCCACCAGGTTCC (reverse). Melt curves were used to verify true positives against a background.

### 2.2 Virus isolation in baby hamster kidney cells

Individual tick homogenates from RNA-positive pools were used to attempt isolation in cell culture. Homogenate was incubated in close contact with baby hamster kidney (BHK) cells for 30 min to 1 hour before additional 2 per cent FBS media were added to the flask. Virus-infected cells were incubated until 20–50 per cent cytopathic effect was observed (approximately 4–5 days post-inoculation). Isolations were validated by plaque assay and qRT-PCR.

### 2.3 Whole-genome sequencing and phylogenetic analysis

DTV genome sequencing was performed from RNA obtained from both tick homogenates and culture supernatants, as previously described (Normandin et al. 2020). Briefly, samples underwent treatment with deoxyribonuclease (DNase) (ArcticZymes), single-cycle cDNA synthesis using random primers and Superscript III (Invitrogen), library tagmentation and 16-cycle PCR amplification using Nextera XT (Illumina), and 150-bp paired-end sequencing using an Illumina MiSeq or NextSeq. Reads underwent reference-based assembly to generate a consensus DTV sequence from each sample, using viral-ngs v2.0.21 (https://github.com/broadinstitute/viral-pipelines) and reference HM440559.1.

Full-length DTV sequences were generated from eighteen RNA samples collected in this study, as well as forty-five primary tick RNA samples provided by collaborators, which were collected between 2018 and 2021 in the Northeast USA (Supplementary Table S1). Full-length POWV and DTV sequences were also obtained from twenty-one culture isolates from ticks collected between 1977 and 2008 (Supplementary Table S1). Sequencing and analysis methods were the same for field-collected ticks, ticks provided by collaborators, and culture supernatants.

These eighty-four newly generated sequences were aligned with twenty-four POWV and DTV reference sequences from GenBank, using MAFFT as implemented in Geneious (https://www.geneious.com). Focused alignments were also made for just the DTV sequences (*N* = 91) and just the DTV sequences from the Northeast (*N* = 75). ModelFinder implemented in IQ-TREE v1.6.12 (Nguyen et al. 2015; Kalyaanamoorthy et al. 2017) was used to identify the best substitution model and gamma shape prior, and maximum likelihood (ML) trees were generated with 1,000 ultrafast bootstraps for each alignment. For each ML tree, root-to-tip analysis was performed using TempEst (Rambaut et al. 2016), with the best-fitting root chosen using the correlation function. Moderate-to-strong support for a molecular clock was observed in all three trees, with a correlation coefficient of 0.86 for all POWVs, 0.63 for DTV alone, and 0.66 for the Northeast sublineage of DTV (Supplementary Fig. S1).

Time-scaled DTV phylogenies were constructed using BEAST2 (Bouckaert et al. 2014) with partitions for codon positions 1+2, codon position 3, and untranslated regions (UTR) (Supplementary Data). Model testing was performed to identify the best-fitting molecular clock and tree prior to assessing population size changes. We compared a strict molecular clock, a local random clock, and a relaxed log-normal clock. For all molecular clock models, the prior was set to 3.5E-5 (Pesko et al. 2010) with a uniform distribution and no upper or lower limits. For tree priors, we compared a constant coalescent (CC) population size, the coalescent extended Bayesian skyline (CEBS), and an exponential coalescent (EC) model. The nested sampling method (Maturana et al. 2017) was used to compare models with a sub-chain length of 20,000 and particle counts of 8 for the CC tree prior and 16 particles for CEBS and EC tree priors.

In our analysis of all DTV sequences (*N* = 91), the marginal likelihoods of the strict clock models were higher than those of the log-normal models, with Bayes factor >2.2 (Supplementary Table S2), providing support for the strict clock model (Kass and Raftery 1995; Maturana et al. 2017). Clock models were also compared using generalized stepping-stone sampling, which again provided support for a strict clock model (Bayes factor = 2.5). Among tree priors, we found that the exponential model was the best fit (Supplementary Table S2).

We separately analyzed DTV sequences from the Northeast sublineage (*N* = 75) and identified two models of equally good fit. First, when using a GTR+G4 nucleotide substitution model (to be consistent with the analyses earlier), we found that a relaxed log-normal clock with the CEBS tree prior was the fit best. However, using a TN93 nucleotide substitution model (as suggested by ModelFinder), a strict clock and an exponential growth model fit best. We did not separately analyze DTV sequences from the Midwest sublineage due to the small sample number and limited time range of collection.

To assess the possible change in the population size of the Northeast DTV subpopulation, we used the CEBS model with the parameters described earlier, running the Markov chain Monte Carlo with 300 million steps. The log files were examined using Tracer v.1.7.2 and confirmed to have ESS values of >200 for all parameters (Rambaut et al. 2018). The CEBS plot was visualized using custom R v4.1 scripts (Heled 2015; R Core Team 2018). The maximum-clade credibility tree was built from the files generated by the CEBS model, with 10 per cent burn-in, using TreeAnatator v2.6.6 (Bouckaert et al. 2014). The median number of population changes was represented by the ‘sum(indicators.alltrees)’ parameter.

### 2.4 Deep sequencing and analysis of passaged virus

Fourteen pairs of DTV-positive tick homogenates and their corresponding cell culture-isolated viruses were sequenced to assess DTV consensus sequence changes after isolation in cell culture. Six of these pairs underwent successful deep sequencing of duplicate independent libraries for the analysis of intrasample single-nucleotide variants (iSNVs). Trimmed, quality-filtered .bam files were generated using viral-ngs v2.0.21, and a merged .bam file was generated using samtools v1.10 (Li et al. 2009). Bam files were separated into respective R1 and R2 fastq files using samtools. Reads <25 bases in length and PCR duplicates were removed using fastp v0.23.2 (Chen et al. 2018). For each library, fastqs for both tick-derived and BHK-derived RNAs were aligned to their respective tick-derived consensus sequences using bowtie2 v2.3.5.1 (Langmead and Salzberg 2012) with the following parameters: --local -L 25 -N 1 --gbar 15 --rdg 5,1 --rfg 5,1 --score-min G,30,15 --mp 10. Output sam files were converted into sorted, indexed bam files using samtools, and iSNVs were called using VPhaser-2 v2.0 (Yang et al. 2013) with the following parameters: -ps 100 -ig 20 -dt 0 -a 0.001. Allele information for positions present in both libraries was extracted from the corresponding merged bam file. iSNVs were then filtered for those present at ≥1 per cent allele frequency, and indels were additionally filtered for those with more than two nucleotides in length to reduce spurious iSNV calls. The remaining iSNVs were manually inspected, and any suspected spurious calls were removed. iSNVs were considered spurious if: (1) iSNV only occurred in one position across multiple reads (e.g. only at the leftmost part of reads and never in the middle of reads), (2) iSNV only occurred in one mapping orientation, and (3) iSNV only occurred in combinations with one another suggestive of processing artifact. Shannon’s entropy was calculated as follows:

$$\frac{- \sum \nolimits {\rm frequency}*{\rm ln}\left({\rm frequency} \right)}{\rm sequence\ length}$$

Normal distribution was assessed by the Q–Q plot, and significance was assessed by the Wilcoxon signed-rank test in SPSS. Associated amino acid changes were annotated using custom genome annotator excel files, and nucleotide positions were indexed to the HM440559.1 genome.

## 3. Results

### 3.1 DTV detected in *I. scapularis* ticks collected in 2019

**Figure 1.**
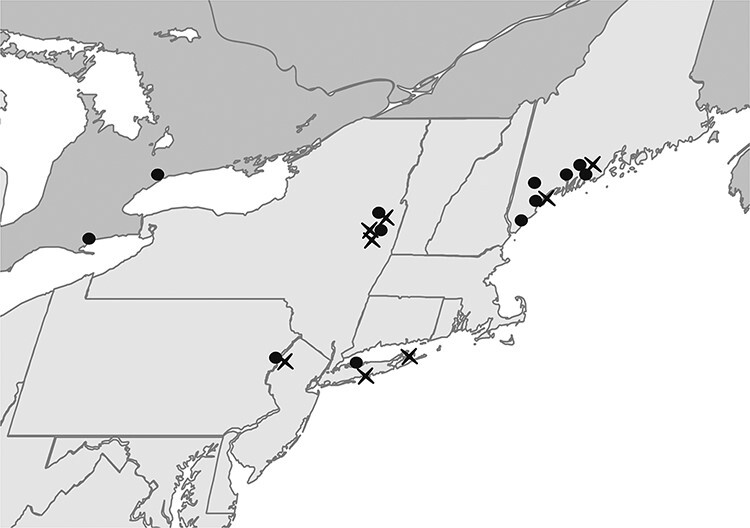
2019 Spring tick collection sites. *I. scapularis* ticks were collected from twenty locations in the Northeast USA (light gray) and Ontario, Canada (dark gray). Powassan-positive locations are indicated by an X. Figure created with biorender.com.

A total of 2,520 *I. scapularis* ticks were collected from twenty locations in the Northeast USA and Ontario, Canada (Fig. 1) in the spring and fall of 2019, the seasonal activity peaks of adult-stage *I. scapularis*. Although *Amblyomma americanum* and *Dermacentor variabilis* ticks were found, no other *Ixodes* species were observed. Because of the seasonal timing of surveys, only eleven *I. scapularis* nymphs were collected, all of which were negative for DTV. Eighteen DTV-positive ticks were detected in eight locations in New York, New Jersey, and Maine. Positive ticks ranged from 1.0 to 5.5 per cent at sites where DTV was detected and fourteen of eighteen positive ticks were female, although the ratio of female to male *I. scapularis* collected was 1.1 (Table 1).

**Table 1.** DTV prevalence in *I. scapularis* by collection location in 2019. For each location, the table lists the number of ticks collected, the number of DTV-positive ticks by qRT-PCR, and, for sites with DTV-positive ticks, the percent of DTV-positive ticks and 95 per cent binomial confidence interval.

Location	Number of *I. scapularis* collected, *n* (ratio F:M)	Number of DTV- positive ticks, *n* (F:M)	Per cent of DTV- positive ticks, *n* (95 per cent CI)
East Stroudsburg, PA	19 (0.7)	0	
Hardwick, NJ	73 (0.9)	4 (3:1)	5.5 (1.5, 13.4)
Lloyd Harbor, NY	22 (1.4)	0	
Cedar Point, NY	71 (0.6)	1 (1:0)	1.4 (0.0, 7.6)
Connetquot, NY	372 (1.5)	4 (3:1)	1.1 (0.3, 2.7)
Saratoga Springs A, NY	101 (1.0)	2 (2:0)	2.0 (0.2, 7.0)
Stillwater, NY	100 (1.5)	0	
Saratoga Springs B, NY	99 (1.2)	2 (2:0)	2.0 (0.3, 7.1)
Saratoga Springs C, NY	124 (1.1)	0	
Saratoga Springs D, NY	60 (2.0)	1 (1:0)	1.7 (0.0, 8.9)
Wells, ME	148 (0.9)	0	
Cape Elizabeth A, ME	84 (2.5)	0	
Cape Elizabeth B, ME	304 (0.7)	3 (1:2)	1.0 (0.2, 2.9)
Standish, ME	113 (0.9)	0	
Newcastle, ME	141 (0.8)	0	
Thomaston, ME	98 (1.7)	0	
Thorndike, ME	12 (1.8)	0	
Rockland, ME	202 (1.2)	1 (1:0)	1.0 (0.0, 2.7)
Rouge Valley, ON	280 (1.1)	0	
Turkey Point, ON	96 (1.5)	0	
Total	2,520 (1.1)	18 (14:4)	0.7 (0.4, 1.1)
Positive sites	1,282 (1.1)	18 (14:4)	1.4 (0.8, 2.2)

### 3.2 Virus population changes after isolation in BHK cells

**Figure 2.**
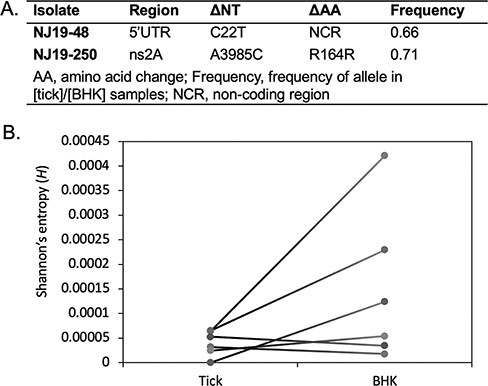
Comparison of DTV population from tick homogenate and virus isolated in cell culture. (A) Consensus-level, SNPs in DTV sequences after one passage in BHK cells. (B) The average Shannon entropy for six paired samples, calculated based on iSNVs from two independent libraries.

Viruses from sixteen of eighteen (88.9 per cent) DTV-positive ticks were isolated in cell culture. Consensus full-length viral genomes were sequenced from fourteen tick homogenates and their corresponding BHK-isolated viruses. For twelve of the homogenate–isolate pairs, the consensus virus sequences were identical. Among the other two, the NJ19-48 isolate differed by one single-nucleotide polymorphism (SNP) in the 5ʹ UTR, and the NY19-250 isolate differed by one synonymous SNP in NS2A (Fig. 2). High-depth sequencing was performed for six tick homogenates and their corresponding BHK-isolated viruses to assess changes in iSNVs and population diversity (Supplementary Table S3). We observed between two and four iSNVs per sample in tick homogenates and between five and forty-two iSNVs per sample in BHK-isolated viruses. While the raw number of iSNVs (not accounting for sequencing depth) increased over one passage for each isolate, the allele frequencies remained mostly under 5 per cent (Supplementary Table S4 and Fig. S2). The average Shannon entropy increased after isolation in cell culture for three of the six isolates (Fig. 2), but not the others (two-tailed Wilcoxon signed-rank test, *P* = 0.156).

### 3.3 Phylogenetic analysis

**Figure 3.**
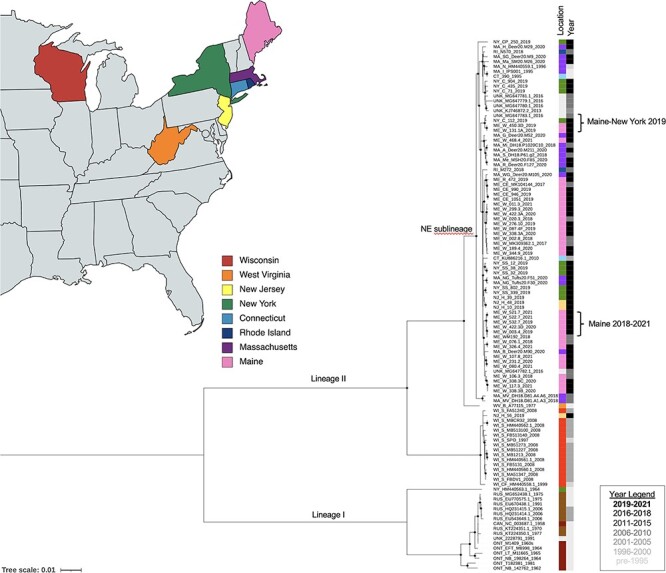
ML phylogenetic tree of 108 POWV genome sequences. Sequence names indicate location, unique identifier, and year. GenBank accession numbers are included for previously reported sequences. Circles indicated nodes with ultrafast bootstrap support of 99 per cent or greater. The location strip plot indicates the US state (with colors matching the map and map legend) or Canada (designated with ‘CAN’), Russia (‘RUS’), or unknown location (‘UNK’). The year strip plot is in grayscale as indicated by the year legend. Labeled branches indicate the two major lineages (I and II) and the Northeast sublineage of the II lineage. Specific clades of interest are marked with brackets. The map was created with mapchart.net.

Our phylogenetic analysis of 108 POWV genome sequences recapitulated the known I and II (DTV) lineages; all sixty-three sequences newly generated from ticks in this study belonged to DTV (Fig. 3). Our analysis also demonstrated the previously described DTV sublineages, one of which contains sequences from the Northeast USA and the other of which contains sequences from the Midwest USA. The average pairwise nucleotide difference was 99.4 per cent between sequences in the Northeast sublineage, 99.7 per cent between sequences in the Midwest sublineage, and 93.6 per cent between sequences from different sublineages. Most of the sequences newly generated in this study clustered with the expected sublineage based on their location of collection. Interestingly, however, a single isolate from New Jersey (NJ-H-56-2019) clustered within the Midwest sublineage and was 99.5 per cent identical to the closest related sequence, WI_FA51240_2008. Sequences from three other samples collected at the same time in the same location (NJ-H-10-2019, NJ-H-39-2019, and NJ-H-48-2019) clustered with the Northeast sublineage and was only 93.5 per cent identical to NJ-H-56-2019.

The Northeast sublineage included samples from multiple locations and years, allowing investigation of population dynamics at a finer scale. We observed clusters of sequences that were geographically restricted but reflected multiple sampling years (e.g. Maine 2018–2021; average pairwise nucleotide difference 99.9 per cent). Other clusters contained closely related sequences collected across multiple locations even within the same year (e.g. Maine—New York 2019; average pairwise nucleotide difference 99.8 per cent). In Connetquot, NY, where *I. scapularis* ticks were abundant, three of four sequences clustered together with 99.9 per cent similarity, while the remaining sequences clustered with sequences from Maine (99.8 per cent identical to sequences from Maine and 98.8 per cent identical to sequences from the same site in New York) (Fig. 3).

### 3.4 Population dynamics

Time-scaled phylogenetic analysis of all ninety-one DTV samples using our best-fitting model, which included a strict molecular clock with the EC tree prior, indicated that sequences within the Northeast sublineage shared a common ancestor approximately 138 years ago (95 per cent highest posterior density (HPD) 88–192) (Supplementary Fig. S3). The most ancestral sequences of the Northeast sublineage were collected from Massachusetts in 2018. Our analysis indicated that the Northeast lineage diverged from the Midwest sublineage approximately 1,184 years ago (95 per cent HPD 770–1,658), and sequences within the Midwest sublineage shared a common ancestor approximately 87 years ago (95 per cent HPD 59–119). The observed clock rate, 6.0 e-5, is somewhat higher than that reported previously for *NS5* alone, 3.9 e-5 (Pesko et al. 2010). Clock rates and marginal likelihoods for models that include a coalescent or CEBS tree prior, as well as log-normal relaxed clock, are shown in Supplementary Table S2.

**Figure 4.**
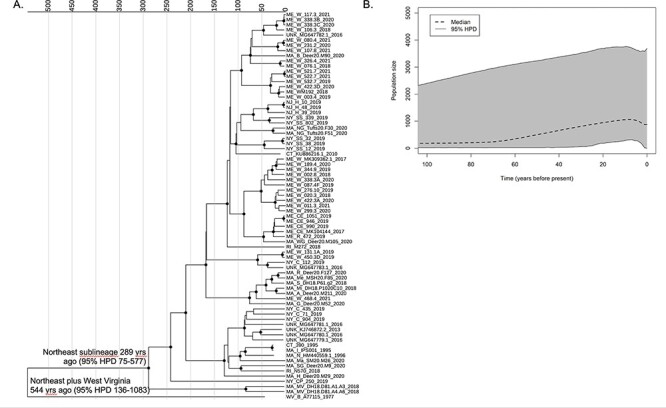
Time-scaled analysis of seventy-five deer tick virus genome sequences from the Northeast USA using a relaxed log-normal clock model and CEBS tree prior. (A) Maximum-clade credibility tree. Sequence names indicate the location, unique identifier, and year. GenBank accession numbers are included for previously reported sequences. Nodes with a posterior probability of 0.95 or higher are marked with circles. Nodes of interest are labeled with their TMRCA (95 per cent HPD). (B) The extended Bayesian skyline plot shows the estimated population size over time since present.

Time-scaled phylogenetic analysis of the seventy-five DTV samples from the Northeast USA was performed using two models with equal fit: a strict clock with EC tree prior and a log-normal relaxed clock with CEBS tree prior. Both yielded similar clock rates (3.2 e-5 and 3.4 e-5, respectively; Supplementary Table S2). The CEBS analysis dated the most recent common ancestor (MRCA) of the Northeast lineage to 289 years ago (95 per cent HPD 75–577) (Fig. 4A) and inferred that there has been at least one change in the DTV population size in the Northeast USA (median 2 changes, 95 per cent HPD 1–3, excluding a constant population size). Plotting population size over time indicated growth over the last 50 years (Fig. 4B). The increase in population size was also supported by posterior estimates of the population growth rate in the exponential population model, which excluded 0 in the 95 per cent HPD interval.

Altogether, our findings suggest that these DTV sequences from the Northeast shared a common ancestor within the past several 100 years and support recent growth in the DTV population size.

## 4. Discussion

We collected, isolated, and sequenced DTV across a wide range of locations and years to increase the understanding of the ecology and evolution of this emerging tick-borne flavivirus. We detected DTV in 1.0–5.5 per cent of ticks screened, in line with previous rates found in Pennsylvania, Connecticut, Maine, and New York (Anderson and Armstrong 2012; Aliota et al. 2014; Robich et al. 2019; Price et al. 2021). The highest DTV prevalence (5.5 per cent) was found in Hardwick, NJ, although only seventy-three ticks were collected in this location. These prevalence data are much lower than those from recent reports of 11–67 per cent in some Pennsylvania counties (Klinepeter 2022). It should be noted that collection methods, confidence intervals, and sample sizes were not reported for these counties, and previous tick surveys in Pennsylvania found around 1 per cent of DTV-positive ticks (Campagnolo et al. 2018; Price et al. 2021). Still, this does not rule out the possibility that focal prevalence could be very high.

We detected significantly more adult female DTV-positive ticks than males, which has not been observed in other tick surveys (Brackney et al. 2011; Robich et al. 2019). The reasons for this are unclear at present but may be related to differences in viral tropisms of DTV between male and female adult *I. scapularis*. For example, tropism for female reproductive tissue, which would be required for efficient vertical transmission, might explain this difference. Alternatively, differences in the size and/or cellular composition of midguts between males and females could alter infection success in ticks (Sonenshine 1991). Thus, understanding flavivirus tissue tropism in both sexes is a critical gap in our knowledge of emerging tick-borne viruses.

We sequenced full viral genomes from eighty-four geographically and temporally diverse samples from ticks and low-passage cell cultures, contributing substantially to the available set of POWV and DTV sequences and allowing us to perform detailed phylogenetic analyses. Our phylogenetic analysis supported the presence of two geographically separated DTV sublineages, consistent with prior reports (Pesko et al. 2010; Anderson and Armstrong 2012; Bondaryuk et al. 2021). Interestingly, a single sample from New Jersey (NJ-H-56-2019) clustered with the Midwest sublineage, suggesting that long-range dispersal of DTV-infected ticks on mammalian or avian hosts is likely, and highlighting the importance of further investigation of DTV in locations situated geographically between the Northeast and Upper Midwest USA. Finer-scale analysis of multiple tick collections in the Northeast from 2017 to 2021 also supported geographic dispersal of closely related viruses over smaller distances, e.g. between sites in Maine and New York, Massachusetts and Maine, and New Jersey and New York. However, we also observed stable foci, in which DTV populations remained highly conserved over time in Maine and Massachusetts. The observation of stable foci in this study, which used an expanded sample set and full-genome sequencing, bolsters findings from prior studies that also identified stable foci of infection using partial sequences and fewer collection sites, which may have limited observations of intermingling between sites (Brackney et al. 2008; Anderson and Armstrong 2012).

Time-scaled phylogenetic analysis of DTV sequences indicated that the Northeast and Midwest sublineages last shared an MRCA approximately 1,200 years ago. Our results (time to most recent common ancestor (TMRCA) 1,184 years ago, 95 per cent HPD 770–1,658) were consistent with the results from an analysis of twenty-six full-genome sequences in 2019 (TMRCA 1,160 years ago, 95 per cent HPD 760–1,750) (Bondaryuk et al. 2021). These recent analyses provide an update to prior studies which had available only partial genome sequences from a smaller number of samples (Pesko et al. 2010). While the Northeast and Midwest lineages diverged from one another over 1,000 years ago, the TMRCA of sequences within each sublineage is just within the last several 100 years. We estimated the TMRCA of the Northeast lineage to be 289 years ago (95 per cent HPD 75–577) when analyzing samples from the Northeast only, and to be slightly more recent but within the same range (138 years ago, 95 per cent HPD 88–192) when analyzing all DTV samples. This timing coincides with reforestation of the USA and booming populations of white-tailed deer, which allowed previously scattered relic populations of *I. scapularis* ticks to disseminate across eastern North America (Sonenshine 2018).

Our results suggest that there has been expansion of the DTV population over the last 50 years. Our inferences of population size must be interpreted cautiously, given the wide 95 per cent HPD and the fact that most of our samples were derived from the recent past. However, DTV population expansion would be consistent with population genetic studies of *I. scapularis* ticks and another pathogen it transmits, *Borrelia burgdorferi*, the spirochete that causes Lyme disease. Both *I. scapularis* and *B. burgdorferi* have experienced rapid population growth within the past 100 years (Qiu et al. 2002; Khatchikian et al. 2015; Xu, Wielstra, and Rich 2020). Tick populations, including *I. scapularis*, have been increasing and expanding northward over the past century due to changes in the landscape, host availability, and climate. Thus, there has been new detection or increased density of ticks and their associated pathogens in territories where little to none were found before (Eisen and Eisen 2018). Further expansion of ticks and tick-borne pathogens is predicted to occur with the ongoing climate change (Bouchard et al. 2019), underscoring the importance of further work to understand the POWV/DTV ecology, evolution, and pathogenesis with the ultimate goal of developing prevention, treatment, and mitigation strategies.

Our study highlights several additional directions of importance. Whereas prior studies of POWV/DTV pathogenesis relied upon a limited number of high-passage viruses, here, we obtained sixteen recent, low-passage isolates from a broad geographic range in the Northeast USA. Successful isolation in cell culture compared to the previous standard of using suckling mouse brain increases the feasibility of isolating viruses for experimental studies. Importantly, we show that isolation in BHK cells does not significantly alter the virus population and rarely produces sequence changes on a consensus level. This observation permits us to conclude that these isolated strains are accurate representations of naturally circulating POWV, indicating their suitability for future studies.

In addition, our study emphasizes the importance of additional collection and sequencing of DTV isolates throughout a broader range of the USA. While we observed geographic dispersal of DTV within the Northeast, additional sequences are needed to determine if this also occurs in the Midwest. Furthermore, sampling of sites between the Northeast and Midwest USA is important for a more comprehensive understanding of DTV dispersal across North America.

In conclusion, we confirm the recent expansion of the DTV population in North America, with geographic patterns of evolution suggesting both geographic dispersal and the repeated establishment of stable foci of infection. These results contribute to our understanding of DTV evolution and ecology in North America.

## Supplementary Material

vead008_Supp

## Data Availability

Newly generated viral sequences in this study are available on National Center for Biotechnology Information (NCBI) GenBank under accession numbers OP823403–OP823484OP823403–OP823484.
