# Supplementary material for: Phylodynamics of deer tick virus in North America
Source: Virus Evol. 2023 Jan 27;9(1):vead008. doi: 10.1093/ve/vead008 (PMC9943884; doi:10.1093/ve/vead008)
Supplement: vead008_Supp [file vead008_supp.zip › suppl_data/POWVPhylodynamics_Supplemental.docx]

**Fig. S1. Root-to-tip analysis of A) all 108 POWV sequences; B) 91 DTV sequences, and C) 75 DTV sequences from the northeast U.S. alone.** The slope for each plot is: 1.26E-4 (A), 9.00E-5 (B), and 2.17E-4 (C).

A)


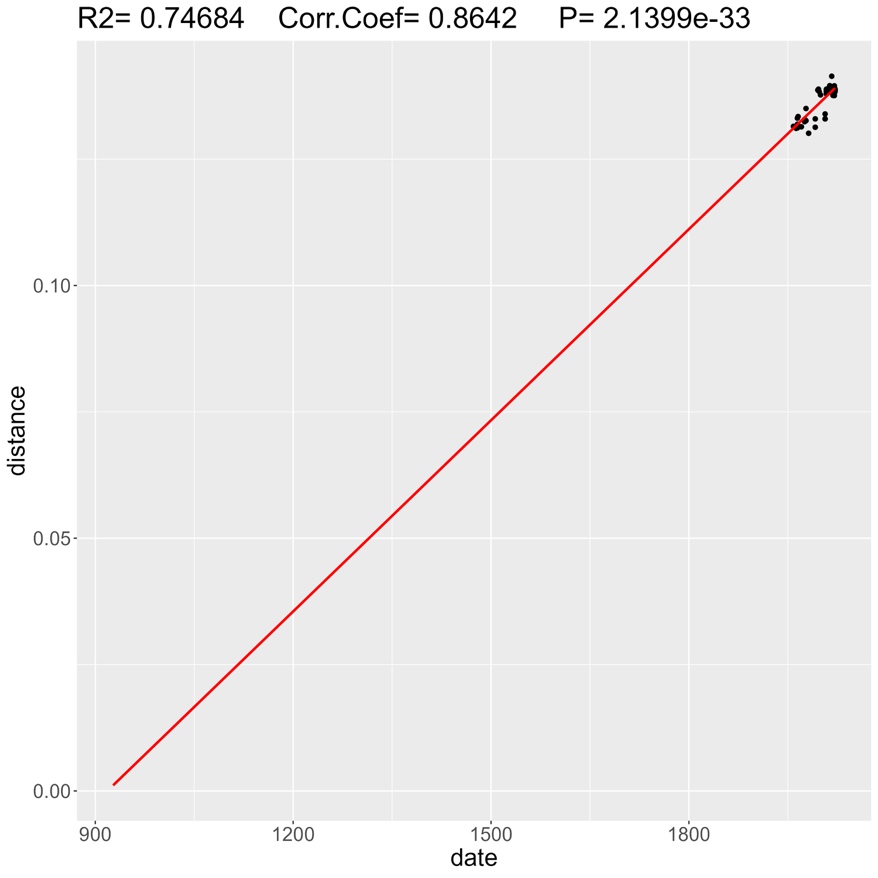


B)


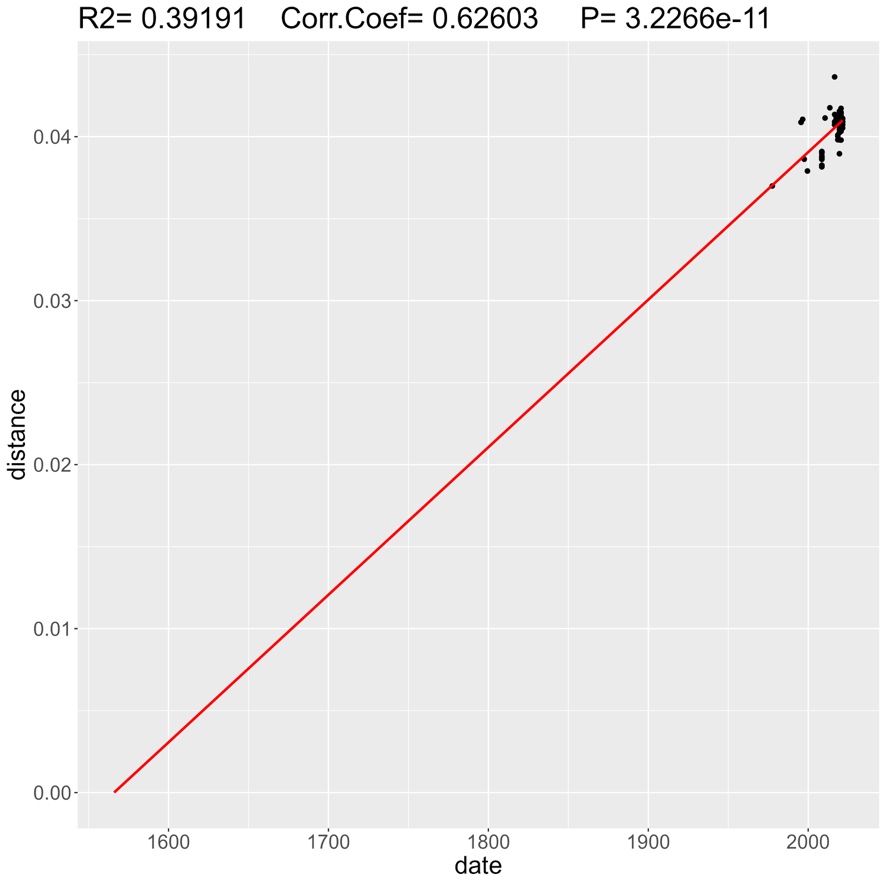


C)


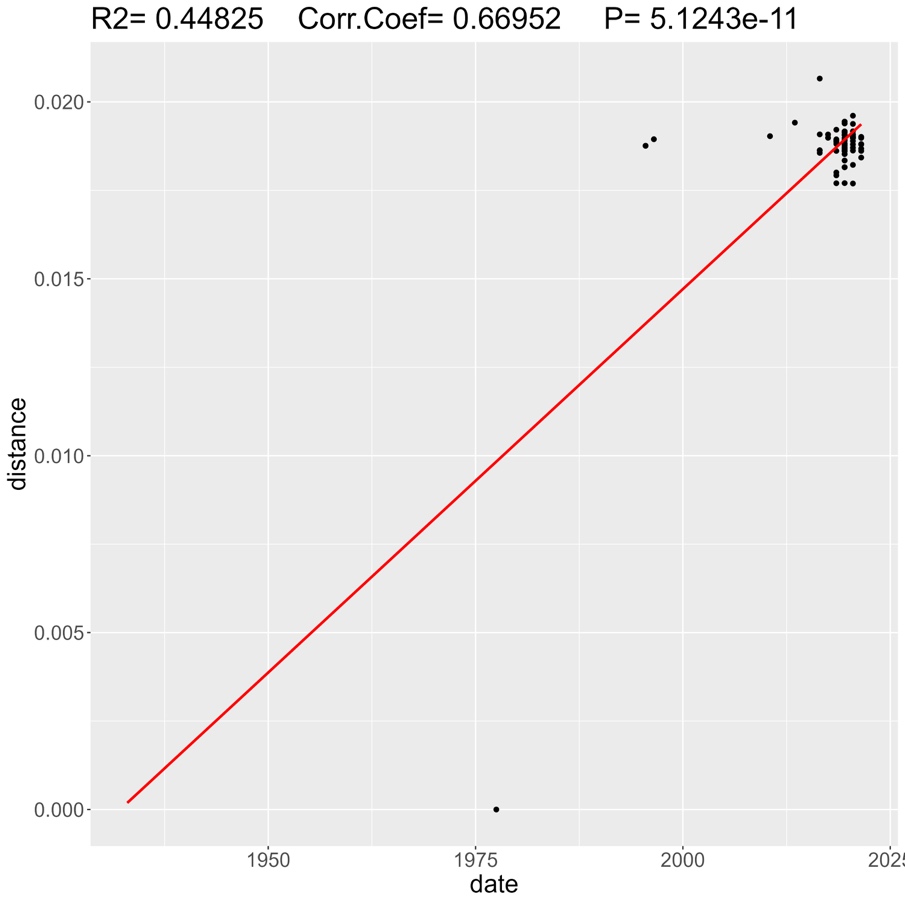


**Fig. S2.** Distribution of iSNV allele frequencies between primary tick isolates (pink) and single passage BHK (blue).


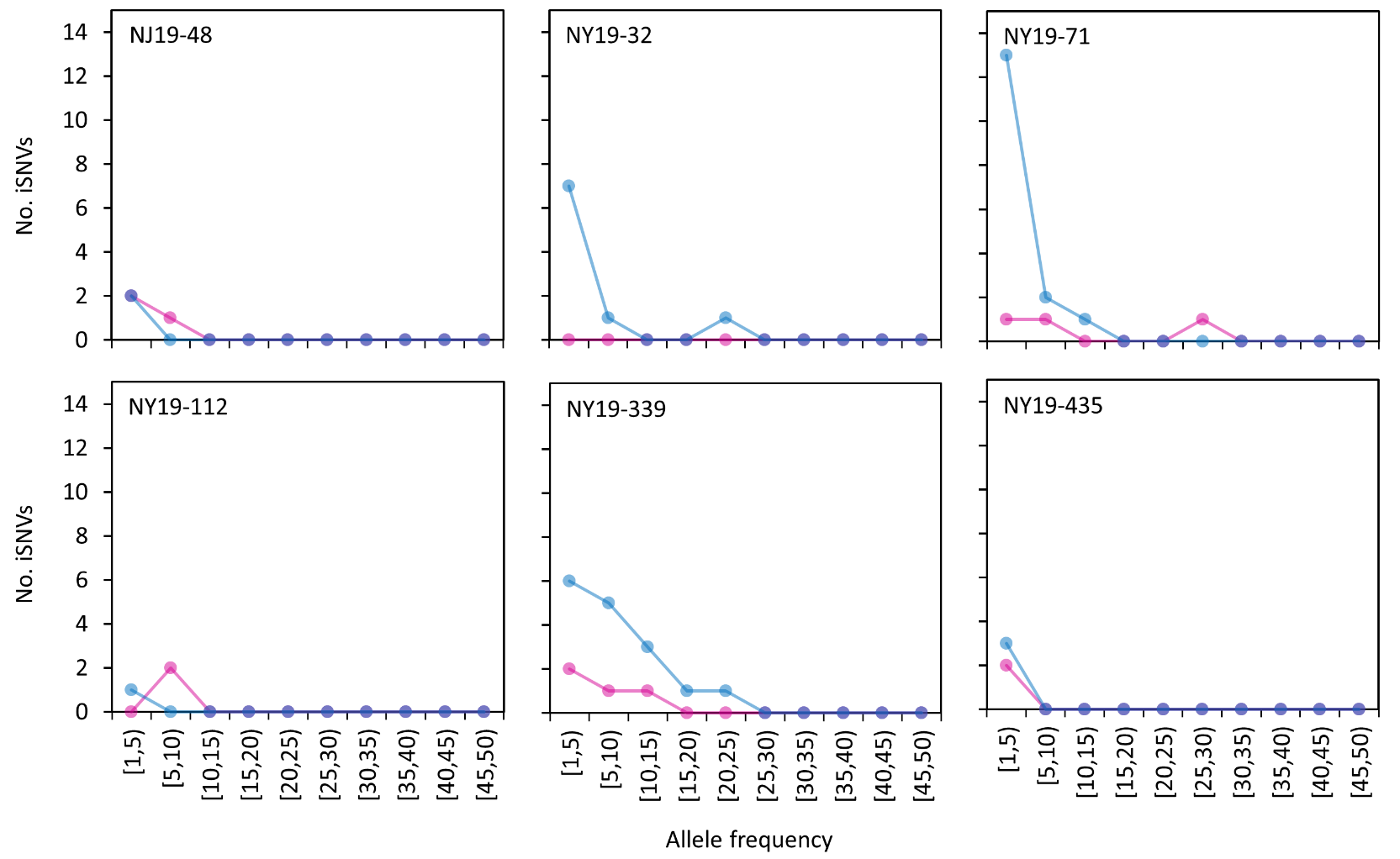


**Fig. S3: Maximum-clade credibility tree of ninety-one Deer Tick Virus genome sequences using a strict clock model and exponential coalescent tree prior.** Sequence names indicate location, unique identifier, and year. GenBank accession numbers are included for previously-reported sequences. Nodes with a posterior probability of 0.95 or higher are marked with circles.

| **Table S1. Sample information and sequencing metrics.** For each sample newly sequenced in this study, the table lists collection date (or year if only year is available), collection location, and passage history. SM: suckling mouse, B: BHK, V: Vero. | | | | | | |
| --- | --- | --- | --- | --- | --- | --- |
| **Sample** | **Collection Date** | **Location** | **Passage history** | **Total Reads** | **Genome Coverage** | **Depth** |
| CT_390_1995 | 1995 | Connecticut | SM1B3V1 | 10,694,347 | 100% | 11,663 |
| MA_A_Deer20.M211_2020 | 2020 | Arena,  MA | 0 | 1,990,630 | 100% | 415 |
| MA_B_Deer20.M90_2020 | 2020 | Bowdoinham,  MA | 0 | 987,547 | 100% | 136 |
| MA_G_Deer20.M52_2020 | 2020 | Glenburn,  MA | 0 | 2,191,421 | 100% | 366 |
| MA_H_Deer20.M29_2020 | 2020 | Harvard,  MA | 0 | 1,077,300 | 100% | 246 |
| MA_I_IPS001_1995 | 1995 | Ipswich,  MA | SM1B2 | 12,237,645 | 100% | 33,819 |
| MA_Ma_SM20.M26_2020 | 2020 | Marshfield,  MA | 0 | 134,806 | 99% | 24 |
| MA_Me_MSH20.F85_2020 | 2020 | Medfield,  MA | 0 | 1,118,040 | 100% | 84 |
| MA_Mi_DH18.P1020C10_2018 | 2018 | Millis,  MA | 0 | 339,653 | 99% | 65 |
| MA_MV_DH18.D81.A1.A3_2018 | 2018 | Martha’s Vineyard, MA | 0 | 317,769 | 99% | 141 |
| MA_MV_DH18.D81.A4.A6_2018 | 2018 | Martha’s Vineyard, MA | 0 | 139,231 | 98% | 97 |
| MA_NG_Tufts20.F30_2020 | 2020 | North Grafton,  MA | 0 | 2,134,002 | 97% | 35 |
| MA_NG_Tufts20.F51_2020 | 2020 | North Grafton,  MA | 0 | 2,104,096 | 100% | 1,064 |
| MA_R_Deer20.F127_2020 | 2020 | Rochester,  MA | 0 | 2,183,464 | 99% | 38 |
| MA_S_DH18.P61.g2_2018 | 2018 | Sherborn,  MA | 0 | 381,416 | 99% | 184 |
| MA_SG_Deer20.M9_2020 | 2020 | South Grafton,  MA | 0 | 242,640 | 100% | 246 |
| MA_WG_Deer20.M105_2020 | 2020 | West Gray,  MA | 0 | 3,501,882 | 100% | 3,397 |
| ME_CE_1051_2019 | 2019-10-30 | Jordan Rd, Cape Elizabeth, ME | 0 | 787,495 | 100% | 2,493 |
| ME_CE_946_2019 | 2019-10-30 | Jordan Rd, Cape Elizabeth, ME | 0 | 803,651 | 100% | 2,195 |
| ME_CE_990_2019 | 2019-10-30 | Jordan Rd, Cape Elizabeth, ME | 0 | 834,072 | 100% | 2,356 |
| ME_R_472_2019 | 2019-05-16 | Oyster River Bog, Rockland, ME | 0 | 104,496,691 | 98% | 25 |
| ME_W_002.8_2018 | 2018-09-27 | Wells,  ME | 0 | 16,489,328 | 98% | 61 |
| ME_W_003.4_2019 | 2019-09-24 | Wells,  ME | 0 | 5,659,209 | 99% | 194 |
| ME_W_011.3_2021 | 2021-04-15 | Wells,  ME | 0 | 1,596,657 | 99% | 651 |
| ME_W_020.3_2018 | 2018-06-07 | Wells,  ME | 0 | 15,528,025 | 99% | 169 |
| ME_W_076.1_2018 | 2018-10-19 | Wells,  ME | 0 | 7,477,408 | 99% | 3,928 |
| ME_W_080.4_2021 | 2021-04-07 | Wells,  ME | 0 | 1,517,187 | 99% | 206 |
| ME_W_087.4F_2019 | 2019-10-09 | Wells,  ME | 0 | 7,276,742 | 98% | 293 |
| ME_W_106.3_2018 | 2018-10-25 | Wells,  ME | 0 | 12,677,074 | 99% | 643 |
| ME_W_107.8_2021 | 2021-04-07 | Wells,  ME | 0 | 1,491,703 | 99% | 52 |
| ME_W_117.3_2021 | 2021-04-23 | Wells,  ME | 0 | 1,563,502 | 99% | 60 |
| ME_W_131.1A_2019 | 2019-10-16 | Wells,  ME | 0 | 13,676,536 | 99% | 893 |
| ME_W_189.4_2020 | 2020-10-15 | Wells,  ME | 0 | 38,213,127 | 99% | 138 |
| ME_W_231.2_2020 | 2020-10-22 | Wells,  ME | 0 | 28,086,588 | 99% | 180 |
| ME_W_276.10_2019 | 2019-10-16 | Wells,  ME | 0 | 5,043,928 | 99% | 457 |
| ME_W_299.3_2020 | 2020-10-08 | Wells,  ME | 0 | 34,309,318 | 99% | 291 |
| ME_W_326.4_2021 | 2021-10-07 | Wells,  ME | 0 | 1,328,936 | 98% | 93 |
| ME_W_338.3A_2020 | 2020-10-20 | Wells,  ME | 0 | 35,296,095 | 99% | 127 |
| ME_W_338.3B_2020 | 2020-10-20 | Wells,  ME | 0 | 33,601,388 | 99% | 342 |
| ME_W_338.3C_2020 | 2020-10-20 | Wells,  ME | 0 | 17,549,357 | 100% | 242 |
| ME_W_344.9_2019 | 2019-06-28 | Wells,  ME | 0 | 10,484,269 | 99% | 210 |
| ME_W_422.3A_2020 | 2020-11-05 | Wells,  ME | 0 | 27,423,922 | 99% | 118 |
| ME_W_422.3D_2020 | 2020-11-05 | Wells,  ME | 0 | 30,026,014 | 99% | 222 |
| ME_W_450.3D_2019 | 2019-10-25 | Wells,  ME | 0 | 7,807,053 | 99% | 140 |
| ME_W_468.4_2021 | 2021-10-28 | Wells,  ME | 0 | 1,365,482 | 96% | 24 |
| ME_W_521.7_2021 | 2021-11-04 | Wells,  ME | 0 | 1,395,931 | 96% | 18 |
| ME_W_522.7_2021 | 2021-11-04 | Wells,  ME | 0 | 1,125,952 | 99% | 360 |
| ME_W_532.7_2019 | 2019-10-25 | Wells,  ME | 0 | 13,672,685 | 99% | 827 |
| ME_WM192_2018 | 2018 | Wells,  ME | 0 | 449,817 | 99% | 455 |
| NJ_H_10_2019 | 2019-05-04 | Millbrook Flatbrook Rd, Hardwick, NJ | 0 | 32,106,046 | 100% | 609 |
| NJ_H_39_2019 | 2019-05-04 | Millbrook Flatbrook Rd, Hardwick, NJ | 0 | 21,699,338 | 99% | 52 |
| NJ_H_48_2019 | 2019-05-04 | Millbrook Flatbrook Rd, Hardwick, NJ | 0 | 36,911,784 | 100% | 628 |
| NJ_H_56_2019 | 2019-05-04 | Millbrook Flatbrook Rd, Hardwick, NJ | 0 | 33,157,057 | 99% | 30 |
| NY_C_112_2019 | 2019-05-02 | Connetquot,  NY | 0 | 591,415 | 100% | 1,100 |
| NY_C_435_2019 | 2019-05-02 | Connetquot,  NY | 0 | 765,182 | 100% | 950 |
| NY_C_71_2019 | 2019-05-02 | Connetquot,  NY | 0 | 342,482 | 100% | 371 |
| NY_C_904_2019 | 2019-05-02 | Connetquot,  NY | 0 | 823,715 | 99% | 11 |
| NY_CP_250_2019 | 2019-05-01 | Cedar Pointe,  NY | 0 | 178,126 | 100% | 642 |
| NY_SS_12_2019 | 2019-05-07 | Saratoga Springs, NY | 0 | 729,095 | 98% | 42 |
| NY_SS_32_2019 | 2019-05-06 | Saratoga Springs, NY | 0 | 356,883 | 99% | 130 |
| NY_SS_339_2019 | 2019-05-06 | Saratoga Springs, NY | 0 | 329,651 | 100% | 308 |
| NY_SS_38_2019 | 2019-05-06 | Saratoga Springs, NY | 0 | 797,322 | 98% | 58 |
| NY_SS_802_2019 | 2019-05-06 | Saratoga Springs, NY | 0 | 448,489 | 100% | 491 |
| ONT_EFT_M8998_1964 | 1964 | E. Ferris township, Ontario | P1SM1V1B1 | 2,151,720 | 100% | 4,512 |
| ONT_LT_M11665_1965 | 1965 | Laurier Township, Ontario | P1SM1V1B1 | 3,617,759 | 100% | 4,187 |
| ONT_M1409_1960s | 1960 | Ontario | P4SM1B1 | 1,667,415 | 100% | 3,996 |
| ONT_NB_142762_1962 | 1962 | North Bay,  Ontario | SM3B1 | 1,909,287 | 100% | 2,329 |
| ONT_NB_198264_1964 | 1964 | North Bay  Ontario | P2V1B1 | 2,022,844 | 100% | 3,110 |
| ONT_T182381_1981 | 1981 | Ontario | P1SM1V1B1 | 1,595,983 | 100% | 3,527 |
| RI_M272_2018 | 2018 | "Trust",  RI | 0 | 413,638 | 97% | 26 |
| RI_N570_2018 | 2018 | "Crew",  RI | 0 | 383,067 | 98% | 67 |
| UNK_2228791_1991 | 1991 | Unknown | P9SM1B1 | 1,578,334 | 100% | 3,410 |
| WI_S_FA51240_2008 | 2008-05-12 | Spooner,  WI | B2 | 10,173,056 | 100% | 34,756 |
| WI_S_FB5131_2008 | 2008-05-13 | Spooner,  WI | B2 | 13,149,650 | 100% | 11,700 |
| WI_S_FB513140_2008 | 2008-05-13 | Spooner,  WI | B2 | 1,291,261 | 99% | 1,413 |
| WI_S_FBDV1_2008 | 2008-05-12 | Spooner,  WI | B2 | 7,546,905 | 100% | 24,009 |
| WI_S_MA51347_2008 | 2008-05-13 | Spooner,  WI | B2 | 8,146,474 | 100% | 10,983 |
| WI_S_MB1213_2008 | 2008-05-13 | Spooner,  WI | B2 | 3,431,284 | 100% | 5,351 |
| WI_S_MB51227_2008 | 2008-05-12 | Spooner,  WI | B2 | 1,642,358 | 100% | 13,966 |
| WI_S_MB51273_2008 | 2008-05-12 | Spooner,  WI | B2 | 2,102,424 | 100% | 4,973 |
| WI_S_MB513100_2008 | 2008-05-13 | Spooner,  WI | B2 | 1,419,789 | 100% | 12,279 |
| WI_S_MBCR32_2008 | 2008 | Spooner,  WI | B2 | 8,451,442 | 100% | 31,104 |
| WI_S_SPO_1997 | 1997 | Spooner,  WI | M1V2B2 | 1,859,908 | 100% | 4,921 |
| WV_B_A77115_1977 | 1977 | Braxton Co, West Virginia | SM3B1 | 1,420,756 | 100% | 10,622 |

**Table S2. Model testing results.** Best-fitting models for each analysis are in bold. CEBS: Coalescent Extended Bayesian Skyline; NS: Nested Sampling

| **Substitution Model** | **Clock Model** | **Tree Prior** | **Mean Clock Rate** | **Clock Rate 95% HPD Interval** | **Growth Rate** | **NS Marginal Likelihood** | **NS Standard Deviation** |
| --- | --- | --- | --- | --- | --- | --- | --- |
| All DTV | | | | | | | |
| GTR+G | Strict | Constant Coalescent | 5.8 E-05 | 3.6 E-5, 8.2 E-5 |  | -26964.5 | 7.9 |
| GTR+G | Lognormal | Constant Coalescent | 7.6 E-05 | 4.6 E-5, 1.1 E-4 |  | -26996.0 | 8.1 |
| GTR+G | Strict | CEBS | 5.2 E-05 | 2.6 E-5, 7.7 E-5 |  | -26978.6 | 5.4 |
| GTR+G | Lognormal | CEBS | 6.7 E-05 | 3.4 E-5, 1.0 E-4 |  | -27014.1 | 6.0 |
| **GTR+G** | **Strict** | **Exponential Coalescent** | **6.0 E-05** | **4.0 E-5, 8.4 E-5** | **4.1 E-04** | **-26963.7** | **5.7** |
| GTR+G | Lognormal | Exponential Coalescent | 8.1 E-05 | 4.9 E-5, 1.2 E-4 | 1.1 E-03 | -26991.0 | 5.7 |
| Northeast DTV | | | | | | | |
| TN93 | Strict | Constant Coalescent | 3.4 E-05 | 1.2 E-5, 5.4 E-5 |  | -27543.9 | 5.3 |
| TN93 | Lognormal | Constant Coalescent | 4.3 E-05 | 1.7 E-5, 7.2 E-5 |  | -23080.3 | 4.9 |
| TN93 | Strict | CEBS | 2.7 E-05 | 4.1 E-6, 4.9 E-5 |  | -23075.3 | 5.1 |
| TN93 | Lognormal | CEBS | 3.4 E-05 | 6.5 E-6, 6.4 E-5 |  | -23089 | 4.9 |
| **TN93** | **Strict** | **Exponential Coalescent** | **3.2 E-05** | 1.2 E-5, 5.3 E-5 | **1.0 E-2** | **-23074.1** | **5** |
| TN93 | Lognormal | Exponential Coalescent | 4.2 E-05 | 1.5 E-5, 7.1 E-5 | 1.5 E-2 | -23089.7 | 5.2 |
| GTR+G | Strict | CEBS | 2.7 E-05 | 4.2 E-6, 5.0 E-5 |  | -23099.4 | 5.4 |
| **GTR+G** | **Lognormal** | **CEBS** | **3.4 E-05** | 5.6 E-6, 6.3 E-5 |  | **-23074.5** | **5.2** |
| GTR+G | Strict | Exponential Coalescent | Did not converge | | | | |
| GTR+G | Lognormal | Exponential Coalescent | 5.7 E-05 | 3.2 E-5, 8.7 E-5 | 1.7 E-2 | -23080.7 | 5.3 |

| **Table S3. Sequencing metrics** of tick and BHK-isolated viruses. | | | | | |
| --- | --- | --- | --- | --- | --- |
| **Isolate** | **Source** | **Library** | **Raw Reads** | **Mapped** | **AvgNTDep** |
| NJ19-48 | Tick | L1 | 73823568 | 845198 | 7365 |
| NJ19-48 | Tick | L2 | 2960608 | 60028 | 656 |
| NJ19-48 | BHK | L1 | 3477206 | 1669071 | 20640 |
| NJ19-48 | BHK | L2 | 5201464 | 2259573 | 28311 |
| NY19-32 | Tick | L1 | 713766 | 20667 | 242 |
| NY19-32 | Tick | L2 | 871478 | 11250 | 133 |
| NY19-32 | BHK | L1 | 2584880 | 958812 | 11490 |
| NY19-32 | BHK | L2 | 2448432 | 802692 | 9779 |
| NY19-71 | Tick | L1 | 684964 | 17485 | 214 |
| NY19-71 | Tick | L2 | 4399272 | 36425 | 437 |
| NY19-71 | BHK | L1 | 2519946 | 322520 | 3817 |
| NY19-71 | BHK | L2 | 3249430 | 360460 | 4487 |
| NY19-339 | Tick | L1 | 1575562 | 8870 | 116 |
| NY19-339 | Tick | L2 | 11164156 | 30305 | 385 |
| NY19-339 | BHK | L1 | 1919628 | 896407 | 10958 |
| NY19-339 | BHK | L2 | 3658396 | 1443035 | 17675 |
| NY19-435 | Tick | L1 | 1530364 | 72229 | 886 |
| NY19-435 | Tick | L2 | 3009250 | 87284 | 1040 |
| NY19-435 | BHK | L1 | 2218344 | 1006819 | 12333 |
| NY19-435 | BHK | L2 | 3188920 | 1171682 | 14189 |

| **Table S4. Confirmed iSNVs** found in duplicate sequencing libraries. | | | | | | | | | | |
| --- | --- | --- | --- | --- | --- | --- | --- | --- | --- | --- |
| **Isolate** | **Source** | **NT** | **NT-Index** | **Con** | **Variant** | **AF*** | **Type** | **Subtype** | **Region** | **ΔAA** |
| NJ19-48 | Tick | 451 | 462 | C | T | 3.19 | Sub | NS | prM | H24Y |
| NJ19-48 | Tick | 706 | 717 | A | G | 9.28 | Sub | NS | prM | R109G |
| NJ19-48 | Tick | 2543 | 2554 | A | G | 2.94 | Sub | NS | ns1 | D40G |
| NJ19-48 | BHK | 2543 | 2554 | A | G | 3.21 | Sub | NS | ns1 | D40G |
| NJ19-48 | BHK | 5169 | 5180 | T | C | 1.47 | Sub | S | ns3 | P201P |
| NJ19-48 | BHK | 7443 | 7454 | C | T | 1.54 | Sub | S | ns4B | N188N |
| NJ19-48 | BHK | 9009 | 9020 | G | A | 1.15 | Sub | S | ns5 | K458K |
| NJ19-48 | BHK | 9105 | 9116 | A | G | 2.23 | Sub | S | ns5 | L490L |
| NY19-112 | Tick | 715 | 717 | A | G | 6.00 | Sub | NS | prM | R109G |
| NY19-112 | Tick | 10372 | 10374 | T | C | 6.07 | Sub | NCR | 3'UTR | NCR |
| NY19-112 | BHK | 9093 | 9095 | T | C | 1.22 | Sub | S | ns5 | R483R |
| NY19-112 | BHK | 10372 | 10374 | T | C | 4.32 | Sub | NCR | 3'UTR | NCR |
| NY19-32 | BHK | 1123 | 1147 | C | T | 1.34 | Sub | NS | E | T68M |
| NY19-32 | BHK | 2010 | 2034 | A | G | 2.04 | Sub | NS | E | T364A |
| NY19-32 | BHK | 3152 | 3176 | C | T | 2.17 | Sub | S | ns1 | V247V |
| NY19-32 | BHK | 3435 | 3459 | T | A | 3.94 | Sub | NS | ns1 | S342T |
| NY19-32 | BHK | 5704 | 5728 | A | G | 23.02 | Sub | NS | ns3 | Q384R |
| NY19-32 | BHK | 6054 | 6078 | A | G | 2.03 | Sub | NS | ns3 | I501V |
| NY19-32 | BHK | 7442 | 7466 | G | A | 4.97 | Sub | S | ns4B | A192A |
| NY19-32 | BHK | 7550 | 7574 | G | A | 2.70 | Sub | S | ns4B | L228L |
| NY19-32 | BHK | 7801 | 7825 | C | T | 4.61 | Sub | NS | ns5 | A60V |
| NY19-32 | BHK | 8963 | 8987 | A | G | 6.71 | Sub | S | ns5 | Q447Q |
| NY19-339 | Tick | 698 | 717 | A | G | 5.27 | Sub | NS | prM | R109G |
| NY19-339 | Tick | 3031 | 3050 | C | T | 4.25 | Sub | S | ns1 | S205S |
| NY19-339 | Tick | 6595 | 6614 | A | G | 11.11 | Sub | S | ns4A | V57V |
| NY19-339 | Tick | 7417 | 7436 | G | A | 4.89 | Sub | NS | ns4B | M182I |
| NY19-339 | BHK | 857 | 876 | A | G | 1.02 | Sub | NS | prM | M162V |
| NY19-339 | BHK | 1308 | 1327 | T | C | 12.85 | Sub | NS | E | V128A |
| NY19-339 | BHK | 1333 | 1352 | A | G | 7.78 | Sub | S | E | K136K |
| NY19-339 | BHK | 1492 | 1511 | A | G | 1.62 | Sub | S | E | A189A |
| NY19-339 | BHK | 1493 | 1512 | A | G | 1.94 | Sub | NS | E | S190G |
| NY19-339 | BHK | 1524 | 1543 | T | C | 5.77 | Sub | NS | E | M200T |
| NY19-339 | BHK | 1596 | 1615 | C | T | 1.12 | Sub | NS | E | A224V |
| NY19-339 | BHK | 1672 | 1691 | A | G | 13.01 | Sub | S | E | A249A |
| NY19-339 | BHK | 2119 | 2138 | T | C | 1.03 | Sub | S | E | S398S |
| NY19-339 | BHK | 2203 | 2222 | A | G | 1.20 | Sub | S | E | S426S |
| NY19-339 | BHK | 3031 | 3050 | C | T | 15.05 | Sub | S | ns1 | S205S |
| NY19-339 | BHK | 3125 | 3144 | G | A | 1.32 | Sub | NS | ns1 | G237R |
| NY19-339 | BHK | 3148 | 3167 | T | C | 1.95 | Sub | S | ns1 | F244F |
| NY19-339 | BHK | 3272 | 3291 | A | G | 1.46 | Sub | NS | ns1 | S286G |
| NY19-339 | BHK | 3324 | 3343 | C | A | 2.32 | Sub | NS | ns1 | T303N |
| NY19-339 | BHK | 3846 | 3865 | A | G | 9.71 | Sub | NS | ns2A | N124S |
| NY19-339 | BHK | 4664 | 4683 | C | A | 1.04 | Sub | NS | ns3 | H36N |
| NY19-339 | BHK | 5140 | 5159 | C | T | 4.01 | Sub | S | ns3 | I194I |
| NY19-339 | BHK | 5284 | 5303 | A | G | 1.51 | Sub | S | ns3 | Q242Q |
| NY19-339 | BHK | 5434 | 5453 | A | G | 1.61 | Sub | S | ns3 | E292E |
| NY19-339 | BHK | 6007 | 6026 | T | C | 7.68 | Sub | S | ns3 | D483D |
| NY19-339 | BHK | 6460 | 6479 | A | G | 2.22 | Sub | S | ns4A | V12V |
| NY19-339 | BHK | 6595 | 6614 | A | G | 23.40 | Sub | S | ns4A | V57V |
| NY19-339 | BHK | 6689 | 6708 | G | A | 1.26 | Sub | NS | ns4A | V89M |
| NY19-339 | BHK | 6856 | 6875 | A | C | 2.09 | Sub | S | 2K | I18I |
| NY19-339 | BHK | 6916 | 6935 | C | T | 3.87 | Sub | S | ns4B | G15G |
| NY19-339 | BHK | 7362 | 7381 | A | G | 1.35 | Sub | NS | ns4B | K164R |
| NY19-339 | BHK | 7417 | 7436 | G | A | 13.42 | Sub | NS | ns4B | M182I |
| NY19-339 | BHK | 7483 | 7502 | T | G | 1.19 | Sub | S | ns4B | A204A |
| NY19-339 | BHK | 7616 | 7635 | G | A | 1.84 | Sub | NS | ns4B | G249R |
| NY19-339 | BHK | 8442 | 8461 | A | G | 1.98 | Sub | NS | ns5 | K272R |
| NY19-339 | BHK | 8607 | 8626 | A | G | 6.02 | Sub | NS | ns5 | K327R |
| NY19-339 | BHK | 9043 | 9062 | C | T | 1.32 | Sub | S | ns5 | S472S |
| NY19-339 | BHK | 9097 | 9116 | A | G | 1.08 | Sub | S | ns5 | L490L |
| NY19-339 | BHK | 9175 | 9194 | T | G | 1.23 | Sub | S | ns5 | L516L |
| NY19-339 | BHK | 9924 | 9943 | A | G | 1.49 | Sub | NS | ns5 | N766S |
| NY19-339 | BHK | 10027 | 10046 | G | A | 1.95 | Sub | S | ns5 | A800A |
| NY19-339 | BHK | 10372 | 10391 | C | T | 1.49 | Sub | NCR | 3'UTR | NCR |
| NY19-339 | BHK | 10401 | 10420 | G | A | 1.22 | Sub | NCR | 3'UTR | NCR |
| NY19-339 | BHK | 10465 | 10484 | T | C | 1.02 | Sub | NCR | 3'UTR | NCR |
| NY19-339 | BHK | 10474 | 10493 | C | T | 1.77 | Sub | NCR | 3'UTR | NCR |
| NY19-339 | BHK | 10528 | 10547 | C | T | 1.06 | Sub | NCR | 3'UTR | NCR |
| NY19-435 | Tick | 715 | 717 | A | G | 3.40 | Sub | NS | prM | R109G |
| NY19-435 | Tick | 7682 | 7684 | A | G | 4.93 | Sub | NS | ns5 | K13R |
| NY19-435 | BHK | 2090 | 2092 | T | C | 2.34 | Sub | NS | E | I383T |
| NY19-435 | BHK | 4182 | 4184 | A | G | 1.07 | Sub | S | ns2A | R230R |
| NY19-435 | BHK | 5388 | 5390 | T | C | 1.22 | Sub | S | ns3 | T271T |
| NY19-435 | BHK | 7173 | 7175 | G | A | 1.15 | Sub | S | ns4B | V95V |
| NY19-435 | BHK | 7682 | 7684 | A | G | 3.78 | Sub | NS | ns5 | K13R |
| NY19-435 | BHK | 7701 | 7703 | C | T | 1.15 | Sub | S | ns5 | C19C |
| NY19-435 | BHK | 7800 | 7802 | T | C | 1.18 | Sub | S | ns5 | G52G |
| NY19-435 | BHK | 8510 | 8512 | A | T | 1.24 | Sub | NS | ns5 | Y289F |
| NY19-435 | BHK | 8624 | 8626 | A | G | 1.51 | Sub | NS | ns5 | K327R |
| NY19-71 | Tick | 3052 | 3054 | C | T | 8.95 | Sub | NS | ns1 | R207W |
| NY19-71 | Tick | 5805 | 5807 | C | T | 27.72 | Sub | S | ns3 | D410D |
| NY19-71 | Tick | 7682 | 7684 | A | G | 3.89 | Sub | NS | ns5 | K13R |
| NY19-71 | BHK | 850 | 852 | G | A | 3.04 | Sub | NS | prM | A154T |
| NY19-71 | BHK | 913 | 915 | C | T | 3.33 | Sub | S | prM | L175L |
| NY19-71 | BHK | 1183 | 1185 | A | G | 2.72 | Sub | NS | E | T81A |
| NY19-71 | BHK | 1533 | 1535 | T | A | 3.07 | Sub | S | E | T197T |
| NY19-71 | BHK | 1712 | 1714 | T | C | 6.42 | Sub | NS | E | L257P |
| NY19-71 | BHK | 2254 | 2256 | G | A | 5.49 | Sub | NS | E | V438I |
| NY19-71 | BHK | 2869 | 2871 | C | T | 2.06 | Sub | NS | ns1 | P146S |
| NY19-71 | BHK | 3052 | 3054 | C | T | 2.13 | Sub | NS | ns1 | R207W |
| NY19-71 | BHK | 4014 | 4016 | A | G | 2.50 | Sub | NS | ns2A | I174M |
| NY19-71 | BHK | 4588 | 4590 | T | C | 1.32 | Sub | NS | ns3 | F5L |
| NY19-71 | BHK | 4644 | 4646 | C | T | 2.98 | Sub | S | ns3 | G23G |
| NY19-71 | BHK | 5063 | 5065 | A | T | 1.11 | Sub | NS | ns3 | Y163F |
| NY19-71 | BHK | 5123 | 5125 | T | C | 1.50 | Sub | NS | ns3 | I183T |
| NY19-71 | BHK | 5340 | 5342 | C | T | 3.72 | Sub | S | ns3 | N255N |
| NY19-71 | BHK | 5382 | 5384 | C | T | 1.87 | Sub | S | ns3 | H269H |
| NY19-71 | BHK | 5805 | 5807 | C | T | 12.55 | Sub | S | ns3 | D410D |
| NY19-71 | BHK | 6703 | 6705 | G | A | 4.36 | Sub | NS | ns4A | V88M |
| NY19-71 | BHK | 6939 | 6941 | C | T | 2.34 | Sub | S | ns4B | F17F |
| NY19-71 | BHK | 7341 | 7343 | T | C | 1.57 | Sub | S | ns4B | D151D |
| NY19-71 | BHK | 8624 | 8626 | A | G | 2.67 | Sub | NS | ns5 | K327R |
| NY19-71 | BHK | 9366 | 9368 | T | C | 1.10 | Sub | S | ns5 | Y574Y |
| NY19-71 | BHK | 9513 | 9515 | C | T | 1.70 | Sub | S | ns5 | R623R |
| NY19-71 | BHK | 9999 | 10001 | C | T | 3.43 | Sub | S | ns5 | P785P |
| NT, nucleotide; NT-index, nucleotide indexed to reference genome; Con, consensus; AF, allele frequency; ΔAA, amino acid change.  *For tick isolates, based on frequency in merged library, for BHK based on average frequency across three replicates. | | | | | | | | | | |
